# Barriers to the participation of people with psychosocial disability in mental health policy development in South Africa: a qualitative study of perspectives of policy makers, professionals, religious leaders and academics

**DOI:** 10.1186/1472-698X-13-17

**Published:** 2013-03-11

**Authors:** Sharon Kleintjes, Crick Lund, Leslie Swartz

**Affiliations:** 1 Alan J. Flisher Centre for Public Mental Health, Department of Psychiatry and Mental Health, University of Cape Town, Valkenberg Hospital, Observatory, Cape Town, 7935, South Africa; 2 Alan J. Flisher Centre for Public Mental Health, Department of Psychology, Stellenbosch University, Cape Town, South Africa

**Keywords:** Psychosocial disability, Rights, Participation barriers, Policy development, South Africa

## Abstract

**Background:**

This paper outlines stakeholder views on environmental barriers that prevent people who live with psychosocial disability from participating in mental health policy development in South Africa.

**Method:**

Fifty-six semi-structured interviews with national, provincial and local South African mental health stakeholders were conducted between August 2006 and August 2009. Respondents included public sector policy makers, professional regulatory council representatives, and representatives from non-profit organisations (NPOs), disabled people’s organisations (DPOs), mental health interest groups, religious organisations, professional associations, universities and research institutions.

**Results:**

Respondents identified three main environmental barriers to participation in policy development: (a) stigmatization and low priority of mental health, (b) poverty, and (c) ineffective recovery and community supports.

**Conclusion:**

A number of attitudes, practices and structures undermine the equal participation of South Africans with psychosocial disability in society. A human rights paradigm and multi-system approach is required to enable full social engagement by people with psychosocial disability, including their involvement in policy development.

## Background

People with psychosocial disability have historically been marginalised from mainstream society by longstanding prejudicial beliefs about their right to full citizenship and their ability to contribute meaningfully to decisions that have an impact on their lives [1-3].

In this paper, we use the term psychosocial disability to refer to people who have experienced enduring mental and emotional distress which “in interaction with various barriers…hinder their full and effective participation in society on an equal basis with others” [4]. We use this term to indicate our view that barriers to people with psychosocial disability participating in decision-making, are not simply a result of their mental and emotional distress. Rather, barriers arise in substantial part from the way in which the organisation of society tends to limit the personal, social, political and economic power of people with disability, including people with psychosocial disability [5,6].

Prejudicial beliefs about the lack of capacity to make rational and informed decisions has led to infringements of the rights of people with psychosocial disability to participate in political, legal, clinical and personal decisions which concern their lives [2,7-11]. This lack of meaningful involvement in decision-making has been a hallmark of many people’s experience of the mental health system [12-15]. Stigmatising and dehumanising experiences endured by some people with psychosocial disability within this system have led to the development of an alternative peer-based support system for recovery which operates outside of the traditional mental health system [16]. In Africa, for example, the oldest African country level peer-led advocacy organisation for people with psychosocial disability, Mental Health Uganda, was launched in 1999. A dozen small country level organisations have since established themselves in other African countries. Most of these peer-led orgnisations are members of the Pan African Network on People with Psychosocial Disability (PANUSP), a continental level umbrella body launched in Kampala, Uganda in 2005. PANUSP, a regional member of the World Network on Users and Survivors of Psychiatry (WNUSP), recently ratified its constitution at its first congress since its launch in Cape Town, South Africa, in October 2011 (Kleintjes S, Lund C, Swartz L: Organising for self-advocacy in mental health: experiences from 7 African countries, forthcoming).

The work of activists living with psychosocial disability in high income countries over the past 40 years has led to a growing acceptance of the importance of including people with psychosocial disability in decision-making which affects their lives. This has been associated with a reform of the way in which some role players conceptualise and provide support for the recovery of people living with psychosocial disability [3,17-21]. Common areas where people with psychosocial disability have been consulted include treatment, service development and evaluation, education and training, curriculum development and research [22]. While some progress has been made to develop procedural, organisational and political support for participation of people with psychosocial disability in many countries, their participation has been influential but not transforming of mainstream mental health care [23]. Further, their participation in over-arching policy-making processes is still infrequent [24,25], particularly in low and middle-income countries [26]. Members of PANUSP, for example, are still in the early stages of lobbying for their participation in policy development at country level, and PANUSP is yet to make a regional impact on policy processes on the African continent (Kleintjes S, Lund C, Swartz L: Organising for self-advocacy in mental health: experiences from 7 African countries, forthcoming).

South Africa is no exception to the problem of the voices of people with psychosocial disability being under-represented in policy processes. In an earlier article [27], we noted that South Africans with psychosocial disability have had little opportunity to participate in post-apartheid revision of the legislative, policy and service development framework guiding the country’s new democracy, including mental health reforms. They were not consulted in the drafting of the first post-apartheid mental health policy in the influential “White paper for the transformation of the health system in South Africa”, adopted in 1997. A set of national mental health policy guidelines, consistent with the White paper, were also developed and approved in 1997, but were not formally adopted and implemented at the time [28]. Again, consultation of people with lived experience was scant. The Mental Health Care Act, no. 17 of 2002, promulgated in 2004, was based on a lengthy consultation process with a range of stakeholders. Consultation of people with psychosocial disability was attempted through the prominent nongovernmental organisations (NGOs) for mental health, and via consultation of prominent individual advocates with lived experience. However, there were no formal peer-led structures in the country at the time.

In a more recent article [29] we documented the policy priorities of 40 South Africans with psychosocial disability and stressed the importance of listening to the voices of people with psychosocial disability when developing policy. It is also necessary to listen to and understand the views of policy makers and service providers, if sustainable change is to be made possible, particularly given the asymmetric power relationships between these stakeholders and people with psychosocial disability [2,18].

The aim of this particular article is to document barriers to participation of people with psychosocial disability in the development of South Africa’s over-arching national mental health-related government policies and legislation. In particular, we focus on the opinions of a range of stakeholders who have influence over mental health policy development and who are involved in the implementation of services based on these policies. These are policy makers, professionals, representatives of NGOs working in the mental health sector, and religious leaders. These findings are drawn from data collected by the first author from August 2006 to August 2009 as part of the Mental Health and Poverty Project (MHaPP), which focused on mental health policy development and implementation in Ghana, South Africa, Uganda and Zambia [30]. While officially a public health priority in South Africa, mental health has nevertheless enjoyed lower priority relative to other health programmes in public sector resource allocation. In the early 2000s, low political support for mental health was evident for several years in vacant posts in the national directorate for mental health, adversely impacting on public sector policy and service development and implementation at that time [28]. With the appointment of a new national Minister for Health and a new senior public administrator supportive of a mental health agenda, a new national mental health policy for the country was drafted in late 2010 using the South African findings of the MHaPP study reported here and elsewhere. The draft underwent a preliminary public review by departmental stakeholders during 2011. The updated draft was launched for public comment by the Minister of Health at the country’s first national mental health summit in April 2012 [31]. In late 2012, the policy was adopted for implementation by the National Health Council, the highest decision-making body for health in South Africa.

## Method

### Respondents

Semi-structured interviews (SSIs) were conducted with fifty-six (56) purposefully selected respondents to explore key barriers that might have an impact on involvement of people with psychosocial disability in mental health policy development. As this was the first study of this kind in South Africa, a wide range of stakeholders were interviewed to capture a broad range of opinions. Respondents were drawn from sectors with potential impact on mental health policy and service development. An initial list of 79 stakeholders was compiled, targeting heads of various stakeholder organisations known to be involved in mental health related policy development and implementation at national and regional level in the nine provinces of South Africa. Formal permission was obtained from national and provincial government and statutory and nongovernmental structures to interview the targeted officials. Three months were set aside for interviews within the project timeframe, allowing sufficient time to interview 56 of the targeted individuals. We were unable to secure interviews with representatives of some key departments (for example, the Ministry of Health at that time, the Department of Labour, South African Police Force, and several religious denominations) due to bureaucratic delays, and officials’ busy schedules. Nevertheless, as interviews progressed and themes re-emerged, we were satisfied with the degree of data saturation obtained at the conclusion of the interviews. As the interviews were conducted with people throughout South Africa, the majority of interviews were conducted telephonically, in most instances 1- 2 hour interviews per respondent. Respondents included eleven national public sector policy makers from the South African Presidency (1), Departments of Health (3), Education (2) , Social Development (2), Housing (1), Justice and Constitutional Development (1) and Correctional Services (1); six professional regulatory council representatives for nursing (1), social work (1), psychology (1), occupational therapy (1) and medicine (2); twelve provincial health managers and a mental health review board member; nine representatives from non-profit organisations (NPOs), two disabled people’s organisations (DPOs), religious leaders (5), professional guilds (3), universities and research institutions (6) and a regional representative of the World Health Organisation. These respondents were interviewed in their professional roles and were not asked to self-identify as people with experience of mental and emotional distress, or as supporters of family members with psychosocial disability, although a few spontaneously provided this information during the interviews.

### Instrument development

Questions were included in the semi-structured interview guides developed for the broader MHaPP situational analysis, to elicit these stakeholders’ views on the involvement of people with psychosocial disability in policy making. Table 1 provides a sample of topics and questions included in the interview schedules. The interview questions were posed in general terms to allow the respondents to explore each issue from their own perspective, with the interviewer probing and clarifying their line of thought to more fully understand the respondents’ point of view.

**Table 1:** Sample questions from the interview guide for national policy makers

	
1.	What are the main development priorities in this country?
2.	What economic, political and social factors do you think affect health care delivery in this country?
3.	What are the key challenges that face the health system?
4.	How does the general public in this country view mental illness? Have their views changed over time?
5.	How important is mental health for this government compared to other health conditions? Why is that?
6.	How significant do you feel other sectors’ policies and programmes are for mental health?
7.	How well do the mental health policies and laws address the needs of people living in poverty? How can the situation be improved?
8.	Do you know of any NGOs, community groups or patient groups who focus primarily on mental health? Are they involved in developing mental health laws and policies in this country? How can this be improved?
9.	Should mental health care users be consulted in the development of mental health laws and policy? If yes, how should they be brought on board? *(Probe if necessary: government, NPOs, own organisations.) In which way should they be involved?*
10.	What are the most important reasons why mental health laws and policies are not implemented effectively? What can we do to overcome these problems?
11.	Are there any other comments you would like to make about the mental health policies in your country, and in particular, the role of different people and organisations in the policy-making process?

### Data collection and analysis

SK conducted all the interviews in English. Interviews were recorded with the permission of respondents, and transcribed verbatim. A framework analysis approach [32] was used to develop a coding frame for analysis of the transcripts using NVivo 7 qualitative data analysis software. This approach comprises 5 stages: familiarization, developing a coding frame, coding the text, charting or summarising the themes to arrive at a synthesis of the key ideas emerging under each theme, and mapping out the key themes and findings embedded in the coded and summarised data. Transcripts were multi-coded on the basis of coding frame themes, with additional themes added to the frame as determined by the data.

### Research ethics

The scope, purpose and dissemination methods for the research were clearly spelt out in the informed consent forms. All respondents provided written informed consent, and confidentiality was assured by removal of identifying material from transcripts. Ethical clearance was obtained from the Research Ethics Committee of the Faculty of Health Sciences (REC Ref: 323/2008), University of Cape Town.

## Results

### Support for policy participation

A few respondents had difficulty conceptualizing the idea of policy participation by people with psychosocial disability, as it was a novel idea for them. These respondents included two very experienced “recovery-focused” practitioners, who stated that they did not believe people with psychosocial disability should participate in policy development. They felt that it would be too draining on their limited resources, and potentially harmful to their recovery. Two others felt that people with psychosocial disability would not be able to articulate their needs, and that their views would be better represented by their families or others who care for them:

*Respondent (R): …the mental illness people, we cannot talk with them. They will not listen to you… You rather talk to their parents or their sisters or their brothers. So what I can say generally is that we live with them, we accept them as our brothers, our sisters our family, we don’t undermine them.* Rural male, traditional healer.

The above views notwithstanding, the majority of respondents were supportive of the participation of people with psychosocial disability in policy development.

Interviewer (I): …input to…mental health policy development; what is your view around their role there?

*(R): Ja. There is room for them. These people are not mentally ill all the time…* Male, member of the Health Professions Council of South Africa.

These stakeholders, however, felt that there were many barriers to their participation. The three most common barriers to participation in policy development mentioned were: (a) stigmatization and low priority of mental health, (b) poverty, and (c) ineffective recovery and community supports.

### Stigma and low priority of mental health

Most respondents felt that public attitudes toward people with psychosocial disability were generally negative, and spoke of the exclusion and disempowerment which stigma can bring. Respondents’ comments also highlighted the fact that people with psychosocial disability may experience accumulative discrimination on the basis of race, gender, and other socially marginalizing factors.

*(R): In another town (the independent living unit) was closed down because the community rejected the mental health service users of which…the majority…are from the black race.…they don’t want them in the neighbourhood…I think mental health status is coupled with colour…It boils down to being dangerous.* Rural woman, manager, Department of Health.

Negative attitudes and beliefs also permeate policy priorities. The majority of respondents mentioned the low standing and funding of mental health relative to other areas of public sector policy, resulting in little integration of mental health into the policy agendas of key government sectors.

(I): And how should they (people with psychosocial disability) be brought on board?

(R): From an advocacy point of view. You see. We’ll do the rest of the work.

(I): So, they need to just come and bring their views…your office is open to that kind of consultation?

R: Ja, ja…

(I): Ok…I’m just curious about the strategies you use to interact with them?

(R): It’s basically hearings, public hearings.

(I): Public hearings. Ok…I can’t recall ever having a public hearing devoted to mental health issues?

*R-: Ja, there hasn’t been any.* Male, senior official, Office of the Presidency.

Across stakeholder groups, respondents felt that low political support for mental health on the public policy agenda and competition for resources with other higher priority public concerns remain barriers to improved attention to mental health as a public sector priority. Respondents from NPOs, DPOs, as well as policy makers who had risen from the ranks of NPOs and DPOs in particular commented on the fact that the South African government has prioritised disabled people as a target group within its policies, yet people with psychosocial disability continue to be invisible in the implementation of these directives. Within government priorities, stigmatising beliefs may result in discriminatory policies which exclude people with psychosocial disability from available support, as demonstrated by this policy maker’s comment on restrictions to access to housing support by people with psychosocial disability:

(R): If the person meets the criteria (for housing subsidies), then the person can qualify…

(I): And if…they have a mental health problem?

*R: I think it would be said they are not eligible…the issue of contracting – that is the key issue there.* Female national policy maker, Department of Housing.

Negative beliefs such as the above, about the capacity of people with psychosocial disability to make sound decisions, were raised by a large proportion of respondents from different stakeholder groups. Most were of the opinion that people with psychosocial disability have the inherent capacity for policy participation, but were routinely excluded from policy consultations, due to longstanding discriminatory opinions about their capabilities.

*(R): Policy makers are very unaware of the fact that…the voices of service users need to be heard…The voice must come from within.* Urban woman, social worker, mental health NPO.

Some stakeholders also noted that the psychosocial difficulties which can follow mental ill-health can deeply impact on a person’s confidence and belief in his or her abilities to take up their social roles, particularly in the face of the current pessimistic public view of mental disorders. Many felt that rather than lacking capacity for participation, problems arose as a result of lack of skill in engaging in the policy process. A few practitioners who spoke of their willingness to support initiatives to improve policy participation by people with psychosocial disability, including through the provision of skills training, voiced their concern about their own lack of understanding of policy processes and advocacy skills to engage in this work.

*(R): I think you must play an active role in it…the thing is, I don’t know enough about policies and how policies work…we are not all politicians…we are not all policy makers…so the more guidelines you get, the more participation you might get from people.* Rural man, manager of provincial mental health NPO.

#### Impact on policy participation

Prejudicial views and discriminatory practices can make it a daunting task for people with psychosocial disability to step up to the task of contributing to the policy process. Stigma can negatively impact on their confidence in advocating for their own agenda during policy-making processes.

R: It’s a campaign, you have to speak about it, you have to educate people and rally them behind your cause…but the difficulty with this campaign is in order for someone to take you serious, you would need a psychiatrist and a psychologist and those types of people to be…

(I): Why?

*R: That’s just how it works in society. What will they say: Die mense’s mal (translation: These people are mad), why should I listen to them? Why not get the nurses and the psychologists, and the doctors to support that campaign? It’s in their interest as well…As long as the users and professionals agree on the agenda, go with it…You see, if you agree on what you want, cooperate. You can fight about who controls it, and the politics later. And you will.* Urban man, leader, disability sector.

### Poverty

Respondents from several stakeholder groups felt that the links between poverty and psychosocial disability are little known, poorly understood, and a significant contributor to the neglect of affected people’s recovery in South Africa. Several respondents felt that this link should be more clearly built into government policy, and addressed in poverty alleviation programmes.

*(R): It’s not brought out in policies. There should be a greater emphasis because that would also then lead to more structured preventative programmes. We’re always talking about HIV and poverty, for example – that kind of connection you know, has made headlines. Mental health and poverty hasn’t. And that’s what we need to focus on, because once mental health, you know, mental health obviously hasn’t been a priority…in our government’s list of priorities, mental health is not number one and yet that should be, because that can affect a range of other things…The connection, the comparison to HIV and poverty, we need to have that similar kind of status, and then we can help mental health.* Female, senior official, Statutory body for Social Workers.

Several respondents commented on the fact that people with psychosocial disability may not have the resources to invest in their health and recovery, nor might family members be able to adequately care for a family member who has limited opportunity to contribute to the family’s needs.

*(R): There are mentally ill people within the villages that…get the thin edge of the wedge because they’re not participating in tilling the fields, looking after the goats and the cows…they’re very often just locked up in a hut at the back of the village…and neglected.* Male member of Statutory Board, Health Professions Council of South Africa.

Respondents from the NGO and DPO sector in particular felt that it is more difficult for people with psychosocial disability than for most people to enter or remain in the formal job market because of discriminatory practices. Those who are already employed and become disabled run the risk of losing their job or career advancement opportunities, while the newly employed might be offered inferior conditions of employment. This discrimination flies in the face of South Africa’s employment equity legislation which regulates the appointment and reasonable accommodation of people with disabilities, including people with psychosocial disability.

*(R): They find ways of circumventing the law…you have a group of people who are marginalised, are in an almost permanent poverty trap, and that causes exclusion.* Male, national disability policy maker.

Where people are unable to work for an income due to their disability, most respondents felt that access to social grants is essential to support recovery. A few respondents noted that difficulties qualifying for grants are due to a lack of appropriate expertise and tools for assessment of psychosocial disability.

*(R): The expertise you need to be able to have to make these kinds of decisions about whether somebody should get a grant or not…It’s quite subtle.…Every group that we worked with saw this in entirely moral terms…that we’re talking about lazy people. And these included health professionals…that’s a major access thing, that it’s invisible and stigmatised.* Urban man, mental health researcher and academic.

People may also experience difficulty relinquishing the financial aid provided by a social grant. This grant may be their household’s only source of income, with the loss of the grant negatively impacting on their well-being, value and status within their family.

*(R): They have an income which is much higher than what a farm worker gets…it keeps more people alive than the one it is given to…they will be very nice towards the patient while there’s money…but just after the money is finished, there’s a lot of physical abuse again…and they don’t have the self-esteem or the ability to fight for themselves.* Rural man, manager of mental health NPO.

#### Impact on policy participation

Respondents indicated that people with psychosocial disability living in subsistence-based communities necessarily direct their energy toward meeting the basic needs of their families, rather than policy concerns. This has a material impact on the time and resources they may be able to dedicate to advocacy and policy participation. The impact of poverty on policy participation does not only affect the already-poor. Some respondents indicated that people with psychosocial disability who had been economically stable prior to their illness, may experience a downward spiral to impoverishment which can impact on their resources for advocacy and self-representation.

### Ineffective recovery and community support

While the majority of respondents felt that good work has been done in South Africa to transform apartheid legislation, policies and services since South Africa’s first democratic election in 1994, most respondents were concerned about the failure of government to effectively implement these reforms.

*(R): That is where the biggest gap is. The gap between the wonderful legislation, Bill of Rights and…the resourcing, the providing, the infrastructure for people to access services. That is either seriously lacking, or is, in fact, absent.* Urban man, provincial government director general.

The theme of strengthening implementation structures, especially at local level, was echoed with respect to the implementation of mental health laws and policies. Respondents felt that insufficiently available community support reduces the ability of people with psychosocial disability to participate in community life, including policy development.

*(R): What we have said is people must get out of psychiatric institutions and be back in the community…with whom? I’m saying this Act is such a good Act but…the resources to make the implementation viable and see it happen, they are not there.* Male member of statutory board, Health Professions Council of South Africa.

Respondents from all stakeholder groups emphasised the need to improve resources for basic recovery and local community support to enable people with psychosocial disability to resume family, work and community roles as soon as possible. It was emphasized that mobilising resources for community based supports for people with psychosocial disability should be an intersectoral focus, as demonstrated by this respondent who suggested a Department of Transport travel subsidy as an adjunct to disability benefits received by people with psychosocial disability.

*(R): Ja, ja, it will make life easier because we…we have actually found out that, you know, relapses happen because the patient has nothing. She can’t even go to the clinic, though we are saying they must get to the local clinics. But you find that some of those local clinics, they require the patient to commute, you know, using transport. How is the patient going to do that if he doesn’t have even a cent?* Female, provincial mental health programme manager, department of Health.

Mention was also made of the limitations of an individual approach to supporting people with psychosocial disability, and the need to consider widening the scope of benefits they receive to include a family and community perspective. Respondents felt that a targeted programme for family support is necessary within the overall development of mental health supports.

*R: … my feeling is that a person belongs somewhere in a family and that family belongs somewhere in a community. It’s a network. When we give disabled people support we should not only be physically helping them with wheelchairs and food and a nice building. There’s the emotional side, there’s mental support…we have people who work in hospitals who happen to be sisters, nurses, doctors giving these people love, but where is their family? Because everybody belongs somewhere. That person needs to know: I belong to this family.* Male, Elder, Christian Zionist Church of South Africa.

#### Impact on policy participation

Policy makers and practitioners in particular felt that effecting these intersectoral changes to policy directions would enable interested people with psychosocial disability to play a role in mental health policy development and community action.

(I): Who should create awareness about mental health as a priority?

*(R): One, the first, is those that are being affected…because they know their needs…The second is the community organisations or the NGOs, because they are in the community and they are an entry-point…much of government policies have been influenced by what is coming from the community.* Female national policy maker, Department of Housing.

The barriers mentioned above also impact on whether people are included in community initiatives. Generally, the voices of people with psychosocial disability remain invisible, in part due to the lack of accessible, accepting community structures through which they can voice their opinions. Many respondents spoke of the value of support groups and advocacy groups as vehicles through which interested people with psychosocial disability might be reached to participate in policy development.

## Discussion

This paper provides qualitative insights into environmental barriers to the participation of people living with psychosocial disability in mental health-related policy development in South Africa. These barriers were identified by a range of stakeholders who were able to provide unique insights through their experience in the field. Barriers identified in the South African context are consistent with those from many other low and middle income countries, where people living with psychosocial disability are widely stigmatised [33,34] and mental health is often given low policy priority [35]. This low priority contributes to people with psychosocial disability seldom being included in regulatory provisions for socio-economic upliftment. It also leads to inadequate access to effective supports, which can prolong episodes of mental and emotional distress, and can interfere with participation by users in the social, economic and political life of their communities [3] (Kleintjes S, Lund C, Swartz L: Organising for self-advocacy in mental health: experiences from 7 African countries, forthcoming). Further, support to people who experience mental and emotional distress is still largely thought of as a treatment issue for the attention of the health sector [36]. Results, however, reveal that beyond important treatment concerns, other barriers – poverty, stigma and discrimination – are crucial. These act in a self-reinforcing cycle of social, economic and political disadvantage, entrenching affected people’s vocal and material exclusion from society. It also maintains their powerlessness to change their marginalised position [37].

This marginalisation extends to exclusion from meaningful opportunities to transform policy directions which impact on their lives. In an earlier article [27], we suggested legislative, policy, organisational, practitioner and personal strategies to enable people with psychosocial disability to empower themselves to participate in mental health policy development. These strategies are as yet not in place in South Africa, and the organisation of people with psychosocial disability is in its infancy (Kleintjes S, Lund C, Swartz L: Organising for self-advocacy in mental health: experiences from 7 African countries, forthcoming). While these provisions are necessary for the empowerment of people with psychosocial disability, they have not been sufficient to ensure meaningful participation of people with psychosocial disability in overarching policy development, nor have they improved their influence on policy outcomes [24,25]. These provisions are not effective as they are implemented in an environment in which the dominant relational culture permits social acts that limit or violate the rights of people with psychosocial disability. This culture maintains their socio-economic disadvantage by excluding them from the power and social resources that are afforded to most citizens in a socially inclusive society. This exclusion is accompanied by their automatic inclusion within the dominant bio-medical subculture with which society associates mental and emotional distress. Their journey to understanding their experiences and its significance for a meaningful, self-directed life is subsumed under their assigned patient role. Within this illness paradigm, their right and ability to participate in activities outside of their foregrounded role as patient, such as that of policy participant, is called into question.

This paper adds to our previous findings [27] by indicating that broader structural factors, including poverty, lack of policy priority, and stigma are crucial barriers that need to be addressed, in the opinion of a range of South African stakeholders. This finding is supported by other studies which suggest that it is the very socio-cultural framework within which people with psychosocial disability find themselves, which gives rise to their marginalisation in society [5,38,39]. Fundamental changes to the overall social system within which these marginalising factors exist will be needed to create an environment which will enable people to regain and assert the psychological and social power enjoyed by other citizens [5,38].

Mental health policy frameworks in several countries now emphasise the participation of people with psychosocial disability in all decisions related to their lives [40-42]. This is still an emergent perspective in South Africa. At the first national mental health summit convened by the Minister of Health in April 2012, there was active representation of people with psychosocial disability from the South African Federation for Mental Health– supported provincial advocacy groups in the country, and its national body, the South African Mental Health Advocacy Movement (SAMHAM). There was also representation from independent advocacy bodies for people with psychosocial disability, including The Ubuntu Centre, South Africa’s only registered DPO for people with psychosocial disability, as well as from representatives of some of the smaller local advocacy groups within the country. This level of participation was far greater than what occurred during South Africa’s 1997 mental health policy development process, or the 2002 Mental Health Care Act development process [27]. This ministerial summit could provide a tentative start to what can be the building of a rights-focused mental health system in South Africa, with active participation of people with psychosocial disability. The Summit has already borne new fruit in the recent formal approval of a National Mental Health Policy which locates people with psychosocial disability as a central partner to the policy development, implementation and review process. This development is in line with South Africa’s obligations as a signatory to the United Nations Convention on the Rights of Persons with Disabilities. The Convention obligates civil society and other government sectors to work toward aligning societal mores, legal frameworks, national policies, organisational policies and procedural guidelines, professional curricula, clinical practice guides, research foci and funding policies to effect the systemic changes alluded to above [43].

Given the policy commitments South Africa has now made as a country, it will be important for the organisations of people with psychosocial disability, their allies, and institutional rights-monitoring mechanisms in South Africa to lobby for and track the extent to which these commitments are honoured in the actual implementation of the new mental health policy. This is particularly important as respondents to this study lauded the excellent policies developed in South Africa, but expressed grave dissatisfaction with progress in implementation of the policy framework.

The priorities for policy reform and service development are remarkably similar amongst civil society and government voices reflected in this paper. They also resonate with the structural changes highlighted by a group of 40 South Africans with psychosocial disability as necessary for their recovery and citizenship [30]. There appears to be common ground amongst a diversity of South African stakeholders regarding the rights of people with psychosocial disability to full involvement in policy and service reform, and to participation in empathic alliances and enabling partnerships that promote their priorities.

## Conclusion

A number of attitudes, practices and structures undermine the equal participation of South Africans with psychosocial disability in society. A human rights paradigm and multi-system approach is required to enable full social engagement by people with psychosocial disability, including their involvement in policy development.

## Competing interests

The author(s) declare that they have no competing interests.

## Authors’ contributions

SK: study design, instrument development, data collection, analysis, drafting, final draft. CL: input into study design and instrument development, critical review of drafts of paper, approval of final draft. LS: critical review of drafts of paper, approval of final draft. All authors read and approved the final manuscript.

## Pre-publication history

The pre-publication history for this paper can be accessed here:

http://www.biomedcentral.com/1472-698X/13/17/prepub
